# Supplementary material for: Ultrathin, ultralight dual-scale fibrous networks with high-infrared transmittance for high-performance, comfortable and sustainable PM0.3 filter
Source: Nat Commun. 2024 Feb 21;15:1586. doi: 10.1038/s41467-024-45833-8 (PMC10881466; doi:10.1038/s41467-024-45833-8)
Supplement: Supplementary file 1 — Supplementary Information [file 41467_2024_45833_MOESM1_ESM.pdf]

Supplementary Information

Ultrathin, ultralight dual-scale fibrous networks with high-infrared transmittance for high-performance, comfortable and sustainable $PM_{0.3}$ filter

Yuchen Yang [1, 2, 3], Xiangshun Li [3], Zhiyong Zhou [3], Qiaohua Qiu [3, 4], Wenjing Chen [3], Jianying Huang [1, 2], Weilong Cai [1, 2], Xiaohong Qin [3, *], Yuekun Lai [1, 2, *]

[1] *Qingyuan Innovation Laboratory, Quanzhou 362801, P. R. China;*

[2] *College of Chemical Engineering, Fuzhou University, Fuzhou 350116, P. R. China;*

[3] *Key Laboratory of Textile Science & Technology of Ministry of Education, College of Textiles, Donghua University, Shanghai 201620, P. R. China;*

[4] *College of Textile Science and Engineering, Zhejiang Sci-Tech University, Hangzhou 310018, P. R. China*

***Corresponding authors. E-mails:** xhqin@dhu.edu.cn (Xiaohong Qin), yklai@fzu.edu.cn (Yuekun Lai)

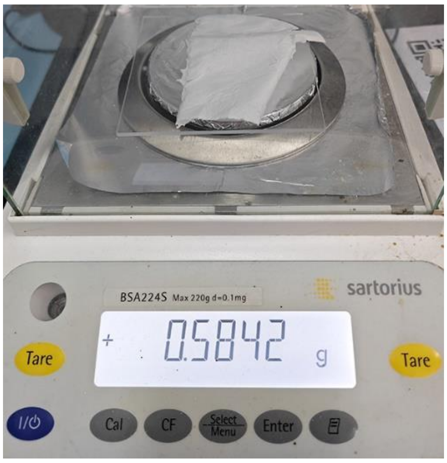

Supplementary Fig. 1. The optical image for verifying the weight of commercial N95 mask.

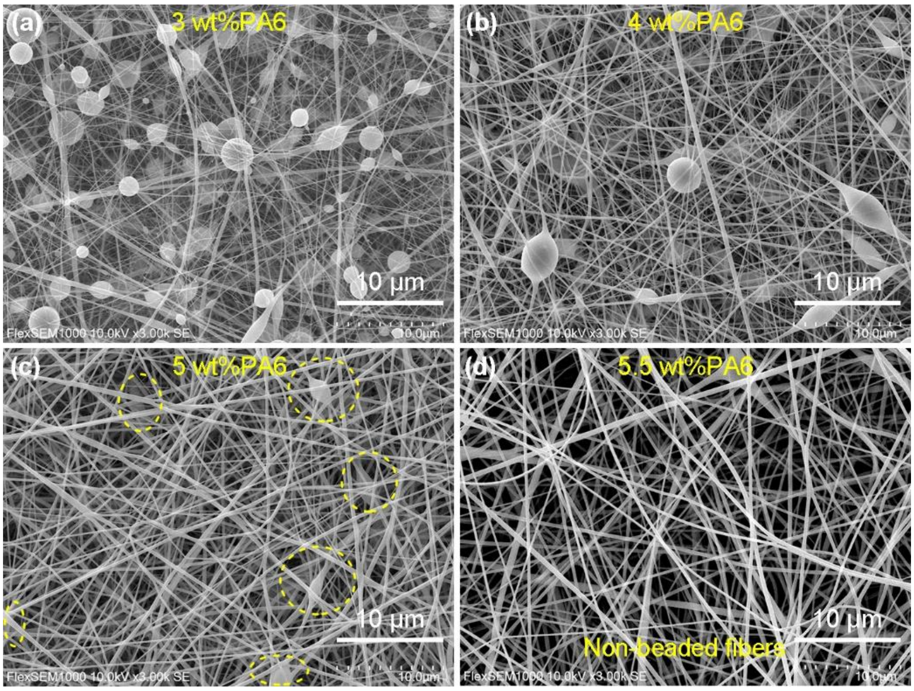

Supplementary Fig. 2. SEM images of PA6 fiber mats produced by (**a**) 3 wt%, (**b**) 4 wt%, (**c**) 5 wt% and (**d**) 5.5 wt% PA6 solutions.

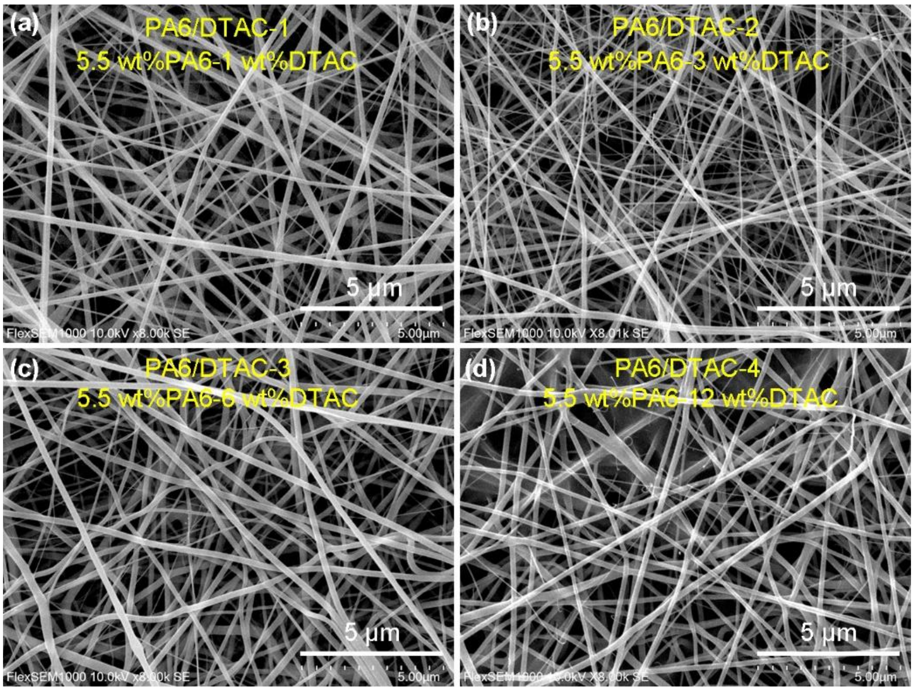

Supplementary Fig. 3. SEM images of PA6/DTAC fiber mats produced by (a) 1 wt%, (b) 3 wt%, (c) 6 wt% and (d) 12 wt% DTAC ratios.

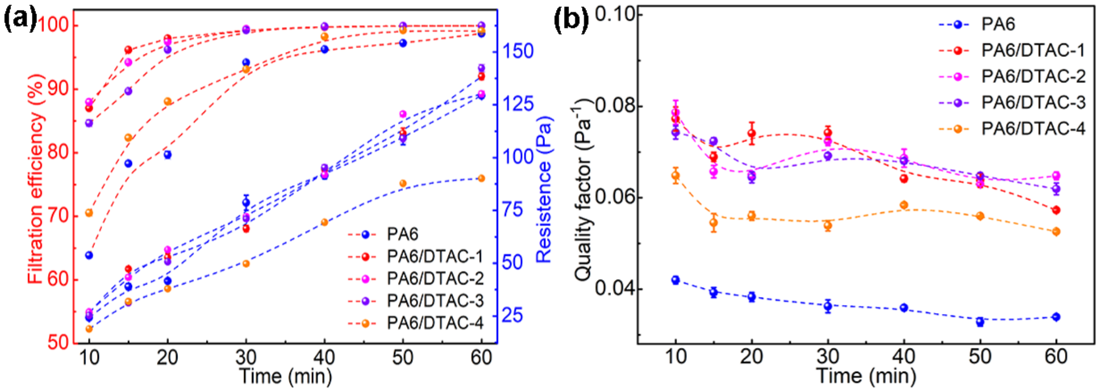

Supplementary Fig. 4. (**a**) Filtration efficiency, filtration resistance and (**b**) quality factor of various PA6 and PA6/DTAC fiber filters with different electrospinning time.

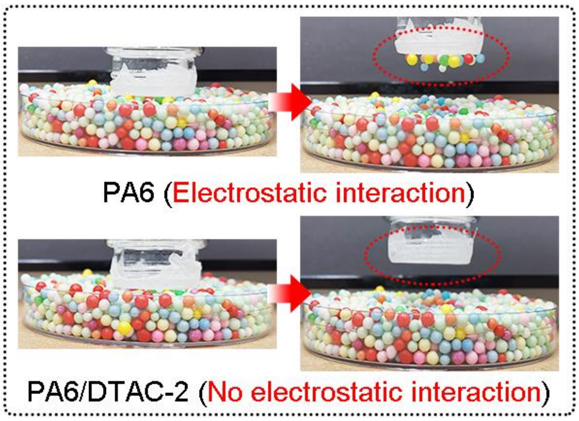

Supplementary Fig. 5. Optical images of PA6 and PA6/DTAC-2 filter attracting foam balls.

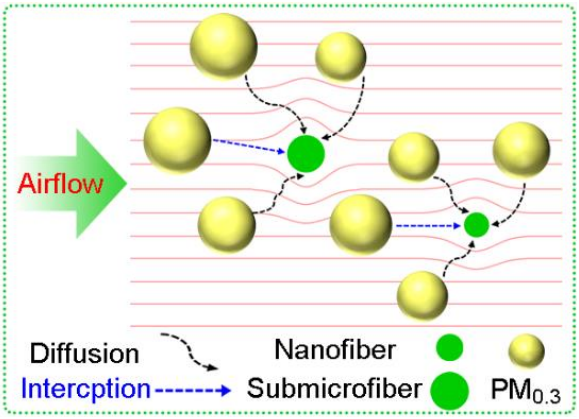

Supplementary Fig. 6. Diagram of $PM_{0.3}$ capture of dual-scale fibrous filter.

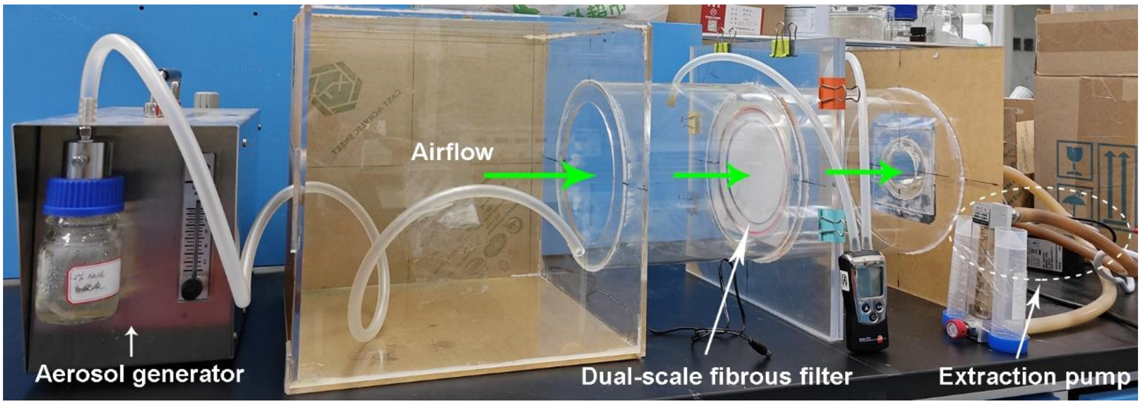

Supplementary Fig. 7. Optical images of self-made apparatus for long-term filtration.

Supplementary Table 1. Filtration performances comparison between our work and those from the recent literatures of Supplementary references.

Supplementary references	Base weight (g/m^2)	Thickness (μm)	PM size (μm)	Airflow (L/min)	Filtration efficiency (%)	Pressure drop (Pa)
[1]	-	-	$PM_{2.5}$	180	99.59	26
[2]	10	-	$PM_{0.3}$	5	97	10
[3]	-	-	$PM_{0.3}$	32	99.97	189
[4]	0.64	-	$PM_{0.3}$	32	99.1	78
[5]	6	20	$PM_{0.3}$	210	98.1	84
[6]	31.2	10.16	$PM_{1.0}$	32	91	91
[7]	-	13	$PM_{0.3}$	32	99.2	90
[8]	-	-	$PM_{0.3}$	32	99.3	127.4
[9]	5.4	-	$PM_{0.3}$	32	96.1	57
[10]	80	517	$PM_{0.3}$	32	99.97	234
[11]	-	3.9	$PM_{0.3}$	32	95	53
Our work	0.24	-	$PM_{0.3}$	32	97.4	56
Our work	0.36	-	$PM_{0.3}$	32	99.5	72
Our work	0.57	1.49	$PM_{0.3}$	32	99.95	120
Our work	0.69	-	$PM_{0.3}$	32	99.98	130

Supplementary Table 2. Preparation parameters for various electrospun fiber mats.

Samples	PA6 Concentration (wt%)	Mass ratio of DTAC to PA6 (wt%)	Voltage (kV)	Liquid supply rate (mm/s)	Distance (cm)
PA6	5.5	0	15 kV	0.05	10
PA6/DTAC-1	5.5	1	20 kV	0.03	10
PA6/DTAC-2	5.5	3	20 kV	0.018	10
PA6/DTAC-3	5.5	6	20 kV	0.014	10
PA6/DTAC-4	5.5	12	20 kV	0.01	10

Supplementary References:

[1] G. Zhang, Q. Zhu, L. Zhang, F. Yong, Z. Zhang, S. Wang, Y. Wang, L. He, G. Tao. High-performance particulate matter including nanoscale particle removal by a self-powered air filter. *Nat. Commun.* 11, 1653 (2020).

[2] Q. Wang, Y. Wei, W. Li, X. Luo, X. Zhang, J. Di, G. Wang, J. Yu. Polarity-dominated stable N97 respirators for airborne virus capture based on nanofibrous membranes. *Angew. Chem. Int. Ed.* 60, 23756-23762 (2021).

[3] F. Wang, Y. Si, J. Yu, B. Ding. Tailoring Nanonets-engineered superflexible nanofibrous aerogels with hierarchical cage-like architecture enables renewable antimicrobial air filtration. *Adv. Funct. Mater.* 31, 2107223 (2021).

[4] Z. Yang, X. Zhang, Z. Qin, H. Li, J. Wang, G. Zeng, C. Liu, J. Long, Y. Zhao, Y. Li, G. Yan. Airflow Synergistic Needleless Electrospinning of instant noodle-like curly nanofibrous membranes for high-efficiency air filtration. *Small* 18, 2107250 (2022).

[5] Z. Peng, J. Shi, X. Xiao, Y. Hong, X. Li, W. Zhang, Y. Cheng, Z. Wang, W. J. Li, J. Chen, M. K. H. Leung, Z. Yang. Self-charging electrostatic face masks leveraging triboelectrification for prolonged air filtration. *Nat. Commun.* 13, 7835 (2022).

[6] T. Le, E. Curry, T. Vinikoor, R. Das, Y. Liu, D. Sheets, K. Tran, C. Hawxhurst, J. Stevens, J. Hancock, O. Bilal, L. Shor, T. Nguyen. Piezoelectric nanofiber membrane for reusable, stable, and highly functional face mask filter with long-term biodegradability. *Adv. Funct. Mater.* 32, 2113040 (2022).

[7] C. Wang, N. Meng, A. Babar, X. Gong, G. Liu, X. Wang, J. Yu, B. Ding. Highly transparent nanofibrous membranes used as transparent masks for efficient $PM_{0.3}$ removal. *ACS Nano* 16, 119-128 (2022).

[8] X. Gong, C. Jin, X. Liu, J. Yu, S. Zhang, B. Ding. Scalable fabrication of electrospun true-nanoscale fiber membranes for effective selective separation. *Nano Lett.* 23, 1044-1051 (2023).

[9] S. Shi, Y. Si, Z. Li, S. Meng, S. Zhang, H. Wu, C. Zhi, W.-F. Io, Y. Ming, D. Wang, B. Fei, H. Huang, J. Hao, J. Hu. An intelligent wearable filtration system for health management. *ACS Nano* 17, 7035-7046 (2023).

[10] Z. Zhou, T. You, D. Wang, Z. Pan, G. Xu, Y. Liang, M. Tang. Conformal build-up of functionalized air filters with improved air cleaning and bioprotective traps. *Adv. Funct. Mater*. 2306777 (2023).

[11] C. Wang, X. Wang, J. Yu, B. Ding. Highly transparent carbon nanofibrous membranes inspired by dragonfly wings. *ACS Nano* 17, 10888-10897 (2023).
